# Role of Bcr1-Activated Genes Hwp1 and Hyr1 in Candida Albicans Oral Mucosal Biofilms and Neutrophil Evasion

**DOI:** 10.1371/journal.pone.0016218

**Published:** 2011-01-25

**Authors:** Prabhat Dwivedi, Angela Thompson, Zhihong Xie, Helena Kashleva, Shantanu Ganguly, Aaron P. Mitchell, Anna Dongari-Bagtzoglou

**Affiliations:** 1 Division of Periodontology, School of Dental Medicine, University of Connecticut, Farmington, Connecticut, United States of America; 2 Department of Microbiology, University of Texas, Houston, Texas, United States of America; 3 Department of Biological Sciences, Carnegie Mellon University, Pittsburgh, Pennsylvania, United States of America; University of Minnesota, United States

## Abstract

Candida albicans triggers recurrent infections of the oropharyngeal mucosa that result from biofilm growth. Prior studies have indicated that the transcription factor Bcr1 regulates biofilm formation in a catheter model, both in vitro and in vivo. We thus hypothesized that Bcr1 plays similar roles in the formation of oral mucosal biofilms and tested this hypothesis in a mouse model of oral infection. We found that a *bcr1/bcr1* mutant did not form significant biofilm on the tongues of immunocompromised mice, in contrast to reference and reconstituted strains that formed pseudomembranes covering most of the tongue dorsal surface. Overexpression of *HWP1*, which specifies an epithelial adhesin that is under the transcriptional control of Bcr1, partly but significantly rescued the *bcr1/bcr1* biofilm phenotype in vivo. Since *HWP1* overexpression only partly reversed the biofilm phenotype, we investigated whether additional mechanisms, besides adhesin down-regulation, were responsible for the reduced virulence of this mutant. We discovered that the *bcr1/bcr1* mutant was more susceptible to damage by human leukocytes when grown on plastic or on the surface of a human oral mucosa tissue analogue. Overexpression of *HYR1*, but not *HWP1*, significantly rescued this phenotype. Furthermore a *hyr1/hyr1* mutant had significantly attenuated virulence in the mouse oral biofilm model of infection. These discoveries show that Bcr1 is critical for mucosal biofilm infection via regulation of epithelial cell adhesin and neutrophil function.

## Introduction

Oral pseudomembranous candidiasis (thrush) is the most prevalent form of Candida infection in patients with weakened or immature immune systems, such as HIV+ children, neonates and patients with malignancies [1], [2], [3]. A resurgence of oral thrush in children was recently reported due to the rising use of inhaled corticosteroids, affecting up to 40% of children after long term treatment [4]. Surprisingly, up to 15% of children with no underlying immune abnormalities present with oral thrush lesions in the pediatric practice [5].

Pseudomembranous candidiasis is one of several clinical forms of Candida infection and has distinct clinical and histopathological characteristics. Clinically this infection presents as white plaques on the oral mucosa, which can be removed by gentle rubbing [6]. These pseudomembranes were recently recognized as archetypal, complex tissue biofilms and were proposed to be responsible for the recalcitrant nature of this infection [7], [8]. Using a mouse model of oral thrush we characterized these biofilms and discovered that they are complex, comprising of yeast, hyphae, commensal bacteria, and neutrophils that form nests within the biofilm mass [9]. Both host and fungal-derived products fill the intercellular spaces, thus forming a supporting biofilm matrix [9]. Although several C. albicans gene products have been implicated in biofilm development on abiotic surfaces [10], [11], [12], [13], [14], [15], information on genes that enable biofilm formation on mucous membranes has only recently begun to emerge [16].

The transcription factor Bcr1 governs biofilm formation in vivo in the catheter, denture and vaginal models [16], [17], [18]. Although Bcr1 is not required for hyphal morphogenesis, it acts as a positive regulator of hyphal-specific adhesins [11], [18]. Manipulation of Bcr1 downstream target genes through mutation and overexpression showed that the surface adhesins Als3 and Hwp1 significantly contribute to biofilm formation in the catheter model. Because biofilm formation on abiotic and biological surfaces may be regulated by similar processes we hypothesized that a *bcr1/bcr1* mutant may also be defective in oral mucosal biofilm development. Using both in vivo and in vitro models we tested the ability of this mutant to form biofilms on the oral mucosa and dissected the specific contribution of Bcr1-regulated genes in this phenotype.

## Results and Discussion

**Figure 1:**
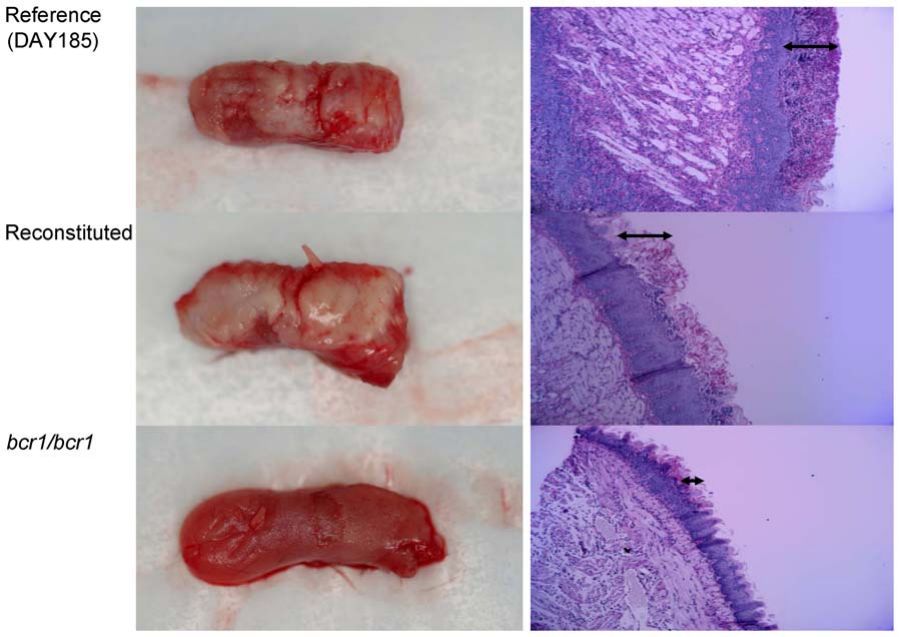
Biofilm formation and histological examination of the tongues of mice infected with the *bcr1/bcr1* mutant, DAY185 (reference) and complemented strains. Tongues of immunocomrpomised animals were excised after five days of infection and the dorsal aspect was digitally photographed. Four mice were infected with each strain and representative clinical pictures are shown from 1 mouse in each group on the left panel. On the right panel, representative PAS-stained thin sections of the tongue of one mouse per group are shown. Arrows indicate the biofilm thickness.

To study the contribution of Bcr1-regulated genes in mucosal biofilms, a mouse oral infection model was used where C. albicans forms white pseudomembranes (biofilms) on the dorsal surface of the tongue [9]. Tongues from animals infected with genetically manipulated strains were excised and examined by macroscopic “clinical” evaluation, assessment of cultivable fungal burden, and histologic analysis to visualize the thickness of biofilms. Consistent with results in the mouse vaginal mucosa model [16], we found that the *bcr1/bcr1* strain was deficient in forming a clinically visible mucosal biofilm on the tongues of immunocompromised mice in vivo (Fig. 1). At the histologic level this mutant formed a thin, interrupted biofilm on the dorsal surface of the tongue (Fig. 1, arrows). These results are in agreement with the recently reported attenuated biofilm phenotype of a *bcr1/bcr1* mutant in the rat denture biofilm model [17].

**Figure 2:**
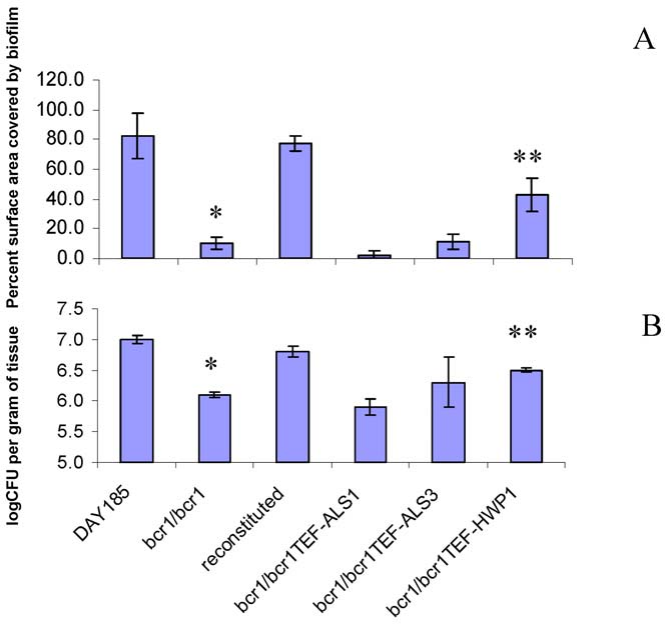
Biofilm surface area and fungal burden of animals infected with the *bcr1/bcr1* mutant and related strains. (A) Percent tongue surface area covered by biofilm. Results represent the average of 4 tongues in each group. Image J was used to calculate the area covered by white plaques as well as the total surface area of each tongue. Error bars represent standard deviations. *p = 0.0000 for *bcr1/bcr1* mutant versus reconstituted strain, **p = 0.026 for *bcr1/bcr1TEF-HWP1* versus *bcr1/bcr1* mutant strain. (B) Tongue fungal burden. Results represent the average of four mice per group and error bars represent standard deviations. *p = 0.0004 for *bcr1/bcr1* mutant versus reconstituted strain, **p = 0.0002 for *bcr1/bcr1TEF-HWP1* versus *bcr1/bcr1* mutant strain.

Surface area estimates of pseudomembranes, examined macroscopically during necropsy, showed approximately 80–100% coverage of the tongue dorsal surface with biofilm formed by the reference and reconstituted strains, while less than 10% of the tongue surface in mice infected with the *bcr1/bcr1* mutant was covered by biofilm (Fig. 2A). In accordance with this we also found that the tongue fungal burden of mice infected with the *bcr1/bcr1* mutant was significantly lower than that of mice infected with either the reference or complemented strains (Fig. 2B).

**Figure 3:**
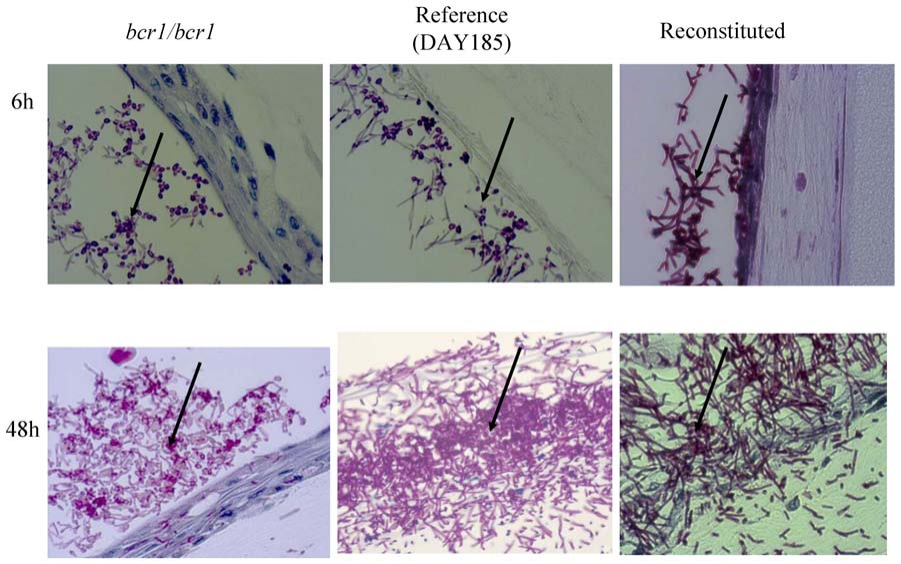
Biofilm formed by the *bcr1/bcr1* mutant, complemented and reference (DAY185) strains on a three-dimensional organotypic model of the oral mucosa. Histologic pictures show 40× magnification after 6 and 24 hours of inoculation. Thin sections were stained with PAS. Arrows indicate biofilms forming on the (apical) epithelial surface of the cultures. Results are representative of one of three experiments.

**Figure 4:**
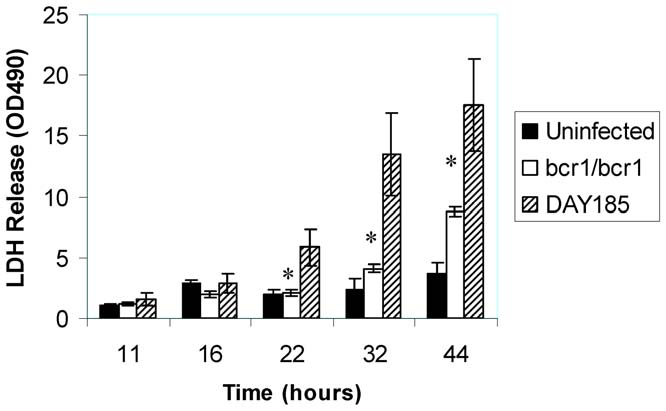
Mucosal damage by the indicated strains in the three dimensional model of the oral mucosa. Cell damage by the *bcr1/bcr1* mutant and reference strains was quantified by the release of lactate dehydrogenase (LDH) in the media. Results are the mean ± SD of three experiments, each condition set up in triplicate. *bcr1/bcr1 mutant significantly different from DAY185 (reference) strain, p = 0.001–0.03.

C. albicans also forms a biofilm when grown on a three-dimensional model of the human oral mucosa [9]. When grown on this model, the *bcr1/bcr1* mutant was slow in forming a mature biofilm, and after 24 hours of growth formed a biofilm comprised mainly of yeast cells, in contrast to its reference and complemented strains (Fig. 3). Moreover, this mutant was significantly less capable of triggering mucosal tissue damage (Fig. 4). This could be attributed to the dominant yeast morphotype in biofilm cells, which lacks expression of several hyphae-specific epithelial adhesins that may also be involved in oral epithelial cell damage [19], [20], [21].

**Figure 5:**
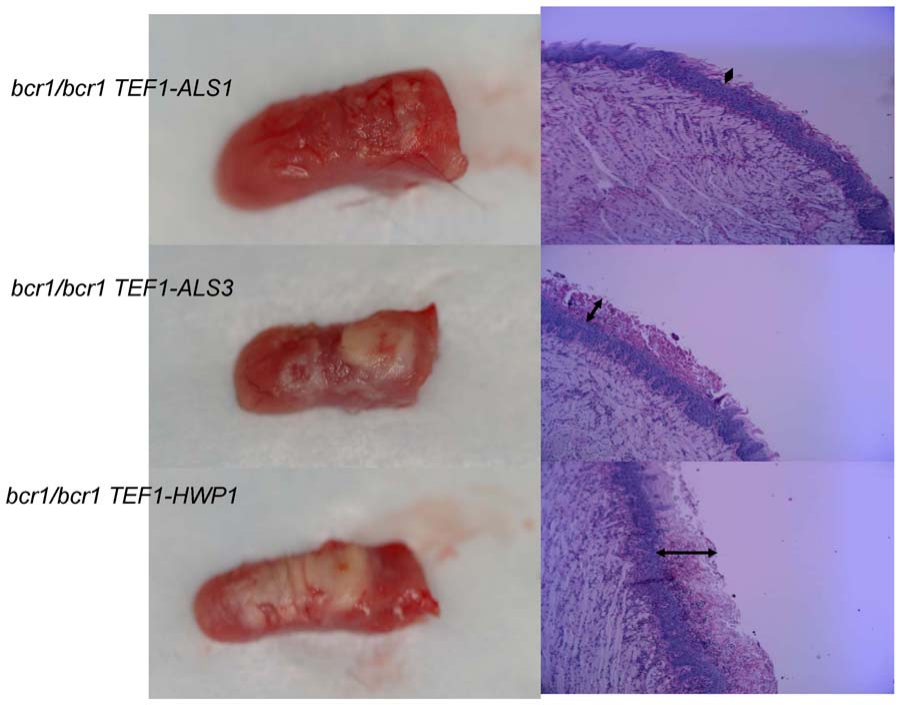
Biofilm formation and histological examination of the tongues of mice infected with strains overexpressing adhesins *ALS1*, *ALS3* and *HWP1* in the *bcr1/bcr1* background. Tongues of immunocomrpomised animals were excised after five days of infection and the dorsal aspect was digitally photographed. Four mice were infected with each strain and representative clinical pictures are shown from 1 mouse in each group on the left panel. On the right panel, representative PAS-stained thin sections of the tongue of one mouse per group are shown. Arrows indicate the biofilm thickness.

Adhesion is a fundamental process under Bcr1 control that promotes biofilm formation on catheter surfaces [18]. Therefore, we evaluated the contribution of the Bcr1-regulated adhesins Als1, Als3 and Hwp1 in the capacity of C. albicans to form a biofilm on the oral mucosa in vivo. Increased expression of *ALS1* in the *bcr1/bcr1* mutant background did not significantly affect the surface area covered by biofilm (Fig. 2A), increase the tongue fungal burden (Fig. 2B), or promote mucosal biofilm formation at the histologic or macroscopic level (Fig. 5). Similarly, expression of *ALS3* under the *TEF1* promoter did not significantly affect the surface area covered by biofilm (Fig. 2A), the tongue fungal burden (Fig. 2B), or the biofilm thickness (Fig. 5, arrows); the strain's intermediate phenotype is discussed further below. A clear example of phenotypic rescue from overexpression was observed with *HWP1*. Specifically, *HWP1* overexpression in this genetic background improved biofilm formation and increased fungal burden in the animals significantly (Fig. 2A, 2B, 5). This finding agrees with observations in the catheter biofilm model where this construct partly but significantly reversed the *bcr1/bcr1* phenotype [18]. In addition to being important in biofilm development [12], [22], Hwp1 is also a well established oral epithelial cell adhesin [20], which plays a role in the pathogenesis of oral infection [23], [24]. Our findings provide additional support for the role of Hwp1 in oral infection, and in addition argue that the reduced expression of *HWP1* in the *bcr1/bcr1* mutant is a major cause of the mutant's oral epithelial biofilm defect.

*ALS3* overexpression in the *bcr1/bcr1* background partly reversed the *bcr1/bcr1* phenotype, based upon visual inspection (Fig. 5). However, the mean percentage surface area covered by biofilm and the CFU counts from infected tissue with this strain did not reach statistical significance (Fig. 2A, 2B). These findings were thus somewhat in contrast with data in the venous catheter biofilm model showing that, although Als3 is not absolutely required for biofilm formation, *ALS3* overexpression completely reverses the biofilm phenotype of the *bcr1/bcr1* mutant [18]. We note that these two biofilm experimental systems are quite different, and that *C. albicans* adhesins exhibit high substrate specificity, even when they have highly related sequences in their binding domains [25]. In addition, innate host defense mechanisms in the oral cavity, coupled with salivary flow and mechanical cleansing by chewing, may modify the ability of these strains to establish a biofilm in vivo.

**Figure 6:**
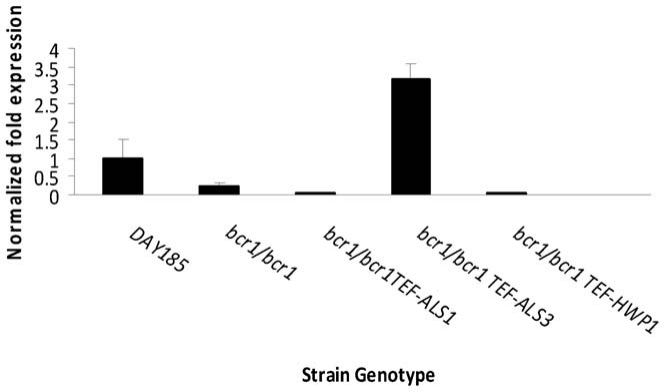
Detection of Als3 expression in the *bcr1/bcr1* mutant, reference (DAY185) and adhesin-overexpressing strains on the 3D model of the human oral mucosa. The indicated strains of C. albicans were grown for 24 h and C. albicans RNA was extracted. The relative transcript levels of Als3 were measured by real-time PCR. Results are the mean ±SD of three biological replicates, each tested in duplicate.

However, because regulation of biofilm-associated gene expression may vary significantly in different biofilm model systems [17], we wanted to rule out the possibility that Als3 expression is Bcr1-independent in the oral mucosa, which could explain the disparate results with these strains in different model systems. Therefore, we quantified Als3 gene expression in the *bcr1/bcr1* mutant and adhesin-overexpressing strains, when grown on a three dimensional model of the human oral mucosa. As anticipated, when grown on an oral mucosa tissue analogue Als3 expression levels were lower in the *bcr1/bcr1* deletion mutant as well as in the *ALS1-* and *HWP1*-overexpressing strains, compared to the reference strain (Fig. 6). In contrast, Als3 expression levels in the *ALS3*-overexpressing strain were three fold higher than the reference strain (Fig. 6). This finding argues against the possibility of differential regulation of Als adhesins in the oral mucosa, and further supports the idea that different experimental systems can reveal tissue-specific functions of adhesins. Thus our results establish that Hwp1, and not Als3, is a critical Brc1 adhesin target relevant to oral thrush. In agreement with our findings, it has been reported that biofilm formation in a subcutaneous rat model requires Bcr1 but not Als3 [26].

Due to the complexity of the structure of oral mucosal biofilms [9], transcriptional deregulation of a single C. albicans gene is unlikely to be responsible for mediating loss of the mucosal biofilm phenotype in the *bcr1/bcr1* mutant. This explains the finding that *HWP1* overexpression only partially reversed the *bcr1/bcr1* phenotype. We thus sought to identify additional, adhesion-unrelated genes under the transcriptional control of Bcr1, contributing to its inability to form mucosal biofilms.

**Figure 7:**
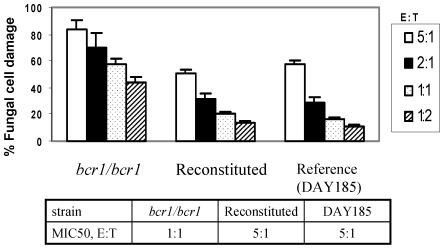
Susceptibility of the *bcr1/bcr1* mutant, reference (DAY185) and reconstituted strains to leukocyte-inflicted damage. Strains were exposed to HL-60 cells that had been differentiated into neutrophil-like cells in vitro. HL-60 cells were added to C. albicans for 3 h at effector to target cell ratios (E∶T) ranging from 5∶1 to 1∶2. Results represent the mean ± SD of three experiments, each condition set up in triplicate.

**Figure 8:**
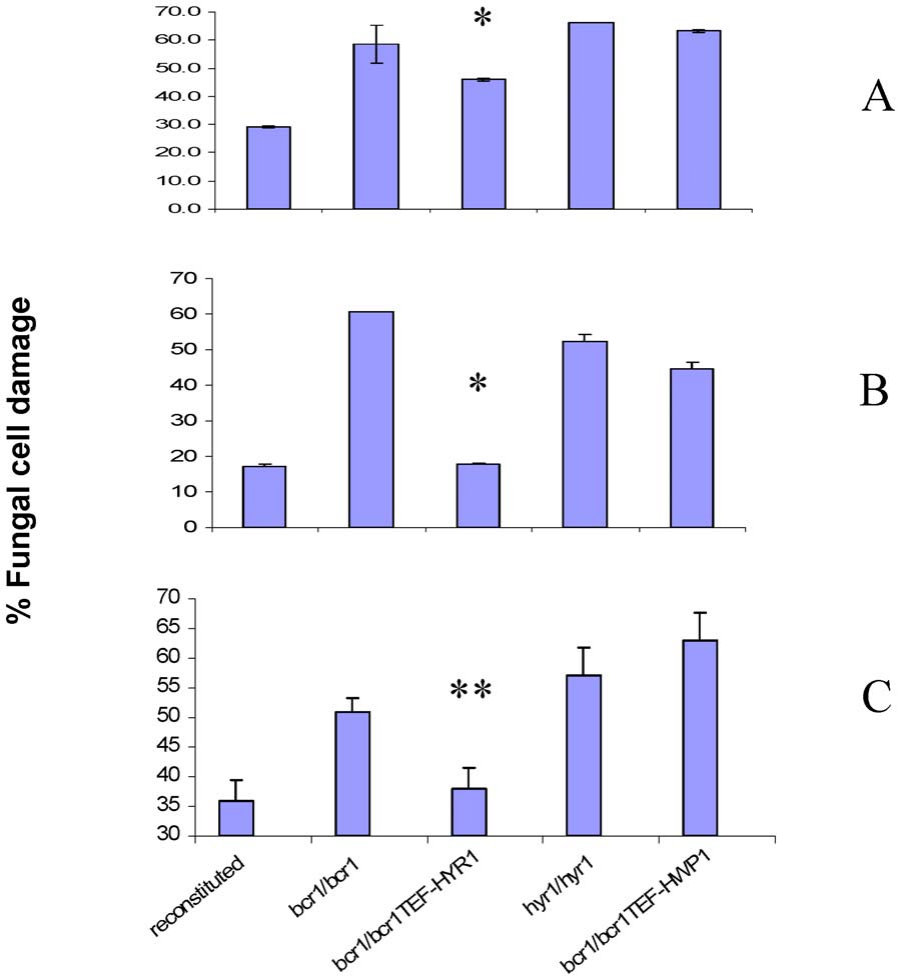
Susceptibility of the *bcr1/bcr1* mutant, *hyr1/hyr1* mutant, and *HYR1-* or *HWP1-* overexpressing strains in the *bcr1/bcr1* background, to human leukocytes. Susceptibility was tested on 96 well plates (A,B) or on a three dimensional model of the human oral mucosa (C). Strains were exposed to differentiated HL-60 cells (A,C) or freshly isolated neutrophils from one human donor (B) at an effector to target cell ratio of 1∶1 (A,B) or 10∶1 (C). Results represent the mean ± SD of three experiments, each condition set up in triplicate. *p<0.03 and **p<0.05 for a comparison between the *bcr1/bcr1* mutant and *HYR1-* overexpressing strain in the *bcr1/bcr1* background.

A prominent feature of infections characterized by soft tissue biofilms is infiltration of infected tissues by neutrophils, which confer innate immune protection [27], [28]. We previously showed that neutrophils infiltrate the oral mucosal biofilm mass in this mouse oral infection model [9]. We thus hypothesized that the *bcr1/bcr1* mutant fails to develop a robust biofilm on the oral mucosa at least partly because it is more efficiently cleared by biofilm-infiltrating neutrophils. To begin to test this hypothesis we examined susceptibility of this mutant to killing on plastic by the HL-60 neutrophil-like cell line using a modification of the XTT assay [29]. Indeed, we found that the *bcr1/bcr1* mutant was more susceptible to killing than its reference and reconstituted strains, regardless of the effector to target ratio used in killing assays (Fig. 7). In fact the effector to target ratio of the *bcr1/bcr1* strain corresponding to the MIC50 in this assay was five times lower than the reference and reconstituted strains (Fig. 7). These findings were confirmed when freshly isolated human neutrophils were used in killing assays (Fig. 8A–B). Finally, we extended these findings to the oral mucosa, by testing susceptibility of the *bcr1/bcr1* mutant to leukocyte killing on a three-dimensional model of the human oral mucosa (Fig. 8C). As expected, this mutant was also more susceptible to leukocyte-inflicted damage when grown on a three dimensional model of the human oral mucosa (Fig. 8C), supporting our hypothesis that it may be more effectively cleared in the oral environment.

Overexpression of *HYR1*, but not *HWP1*, in the *bcr1/bcr1* background, significantly rescued the higher susceptibility phenotype of this mutant to killing by leukocytes both on plastic and on the oral tissue surface (Fig. 8A–C). These findings confirmed previous reports showing that Hyr1, but not Hwp1 is involved in susceptibility to neutrophil killing [30], [31]. More importantly, these data, combined with the finding that the *hyr1/hyr1* mutant was highly susceptible to killing in an oral mucosal tissue model (Fig. 8C), suggest that this gene is indirectly contributing to the observed mucosal biofilm phenotype by conferring resistance to neutrophil killing. Thus, this study now strongly implicates Hyr1 in innate immune cell evasion of *C.* albicans in the oral mucosa.

**Figure 9:**
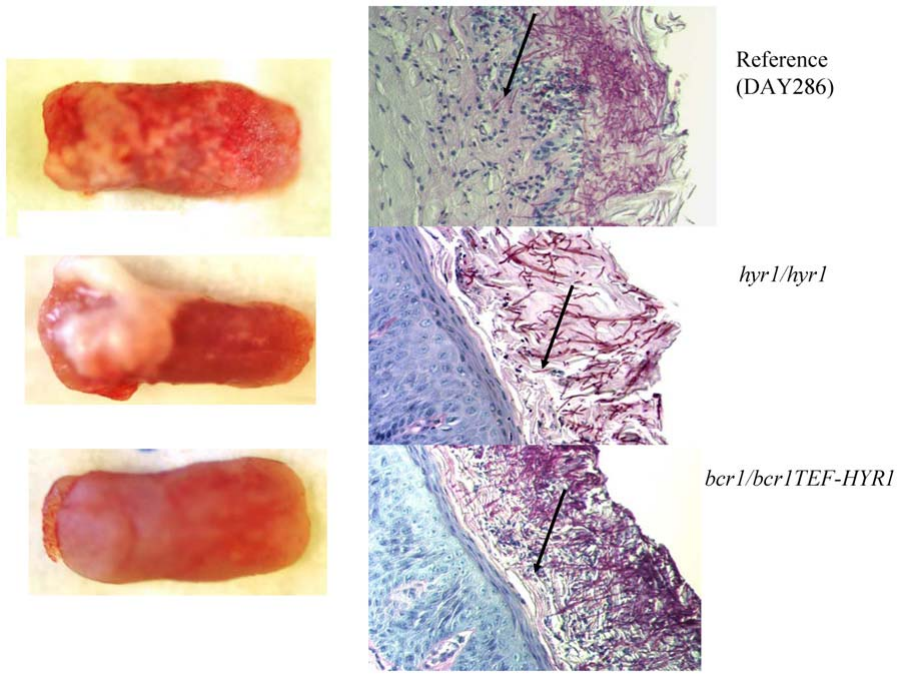
Biofilm formation and histological examination of the tongues of mice infected with DAY286 (reference), *hyr1/hyr1* mutant and *HYR-1* overexpressing strains in the *bcr1/bcr1* background. Tongues of immunocomrpomised animals were excised after five days of infection and the dorsal aspect was digitally photographed. Four mice were infected with each strain and representative clinical pictures are shown from 1 mouse in each group on the left panel. On the right panel, representative PAS-stained thin sections of the tongue of one mouse per group are shown. Arrows indicate microorganisms invading the spinous cell layer of the epithelium (strain DAY286) or remaining superficially within biofilms (*hyr1/hyr1* mutant and *HYR1-* overexpressing strains).

**Figure 10:**
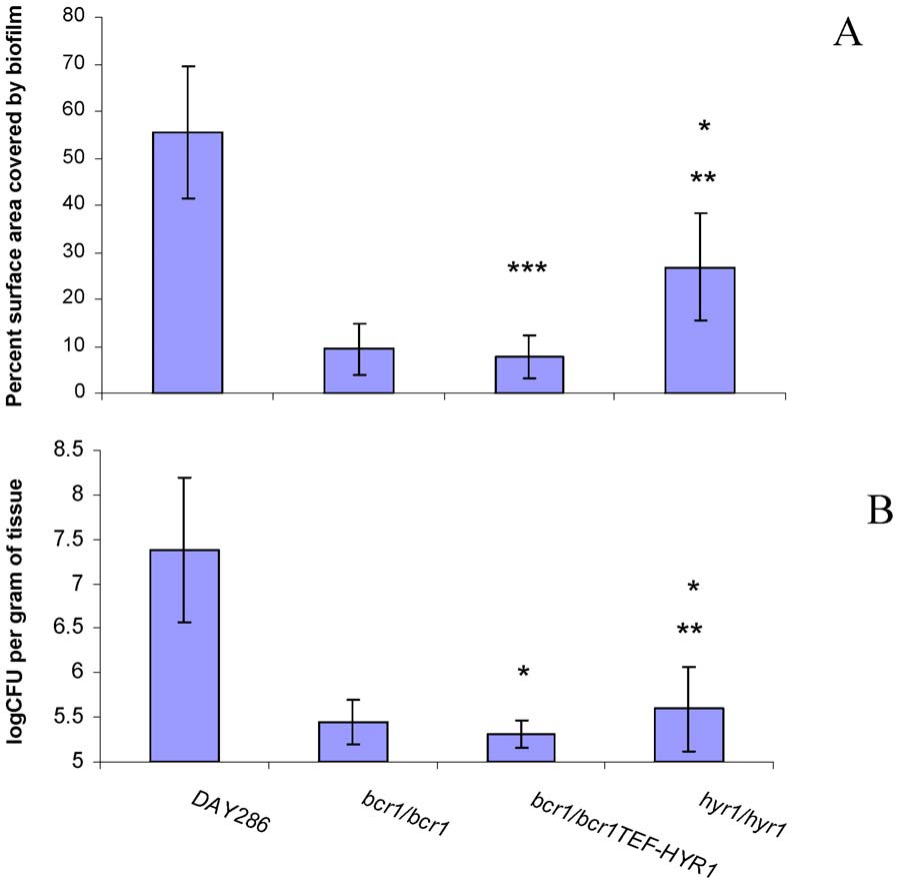
Biofilm surface area and fungal burden in animals infected with the *bcr1/bcr1* mutant, *hyr1/hyr1* mutant, *HYR1-* overexpressing and reference strains. (A) Percent tongue surface area covered by biofilm. Results represent the average of 4 tongues in each group. Image J was used to calculate the area covered by white plaques as well as the total surface area of each tongue. Error bars represent standard deviations. *p = 0.015 for *hyr1/hyr1* mutant versus reference strain; **p = 0.008 for *hyr1/hyr1* mutant versus *bcr1/bcr1TEF-HYR1* strain; ***p = 006 for *bcr1/bcr1TEF-HYR1* versus reference strain. (B) Tongue fungal burden. Results represent the average of four mice per group and error bars represent standard deviations. *p = 0.000 for *bcr1/bcr1TEF-HYR1* or *hyr1/hyr1* compared to reference strain, **p = 0.23 for *hyr1/hyr1* versus the *bcr1/bcr1TEF-HYR1 s*train.

Hyr1 is a GPI-anchored cell wall protein, expressed during hyphal development [32] and repressed upon neutrophil encounter [30], [33], however, relatively little is known about its exact role in virulence. Because there is no severe biofilm defect on catheter surfaces in the *hyr1/hyr1* mutant [18], we hypothesized that this mutant may have only a moderately attenuated biofilm phenotype on the tongue surface. As expected, this mutant formed biofilms covering part of the tongue surface, and overexpression of *HYR1* in the *bcr1/bcr1* background did not rescue the oral mucosal biofilm phenotype (Fig. 9, 10A). However, we also anticipated that due to increased susceptibility to neutrophil killing, preventing deep tissue invasion [34], the tissue fungal burden in mice infected with the *hyr1/hyr1* mutant would be severely attenuated. Consistent with this hypothesis, histological assessment showed that, in contrast to the reference strain which reached the granular and prickle epithelial cell layers, the *hyr/hyr1* mutant was confined within the keratin layer (Fig. 9 arrows). As a result, the tissue fungal burden was severely attenuated in mice infected with the *hyr1/hyr1* mutant (Fig. 10B). However, overexpression of *HYR1* in the *bcr1/bcr1* background was not sufficient to reverse the tissue invasion or fungal burden phenotype of the *bcr1/bcr1* mutant (Fig. 9 arrows, and 10B). This finding is consistent with the fact that, in addition to neutrophil clearance, other virulence mechanisms directly related to epithelial adhesion and invasion, as noted above, are contributing to the severely attenuated phenotype of this mutant.

In conclusion, our studies provide valuable new insights which promote the understanding of the pathogenesis of Candida mucosal biofilm infections. We have shown that the transcription factor Bcr1 is a critical regulator of oral mucosal biofilm formation and identified two genes, *HWP1* and *HYR1*, under Bcr1 control that govern the inability of the *bcr1/bcr1* strain to form robust oral mucosal biofilms. Although much is known about the function of Hwp1 and its role in oral mucosal infection, this is the first study implicating Hyr1 in the pathogenesis of oral biofilm infection.

## Methods

### Ethics Statement

The study was approved by the University of Connecticut Health Center Animal Care Committee (Protocol Number: 2009-541) and the Human Subjects Protection Office (IRB Number: 02-288-2). Animals were monitored daily for distress. Given that the oral cavity is readily accessible, lesions are detected relatively early in their onset and animals are euthanized after lesion formation before visible distress/behavior signs are observed. A written informed consent was signed by all healthy human blood donors.

### C. albicans strains

The C. albicans deletion mutants and overexpressing strains and their construction are described in detail elsewhere [11], [18]. Strain DAY185 is a His^+^ reference strain used to construct the His^+^ *bcr1/bcr1* deletion mutant used in all experiments [18], [35]. Strain DAY286 [36] was used as a reference strain for the *hyr1/hyr1* null mutant in some experiments. The two reference strains were phenotypically similar in all experimental systems described in this study. All strains exhibited similar growth characteristics when grown overnight in epithelial cell media (not shown).

### Mouse model of mucosal biofilm

Different strains were tested for their ability to form an oral biofilm using our previously described mouse model [9]. Briefly, 6–8 week old female C57BL/6 mice were immunosuppressed by subcutaneous injection with cortisone acetate (225 mg/kg, dissolved in 200 µl PBS containing 0.5% Tween-20) on days -1, 1 and 3 relative to infection. To deliver the C. albicans challenge mice were anaesthetized by an intramuscular injection of ketamine and xylazine (90–100 mg/kg and 10 mg/kg of body weight, respectively) and a small cotton pad soaked with 100 µl of C. albicans cell suspension (6×10^8^ cells/ml) was used to swab the entire oral cavity. The swab was left for 2 h under the tongue and was removed before the animals awoke. This procedure was repeated 2 days later and mice were sacrificed after 5 days of total exposure to C. albicans. During the infection period animals were also given drinking water containing a daily-fresh suspension of each strain (6×10^6^ yeast organisms/ml) to maintain high oral carriage loads throughout the experimental period. Tongues were removed aseptically at necropsy, photographed, and images were saved as jpg files. Images were subsequently analyzed using the NIH Image J software (http://rsb.info.nih.gov/ij) and data were expressed as percent surface area covered by biofilm (total surface area of white lesions/entire tongue dorsal surface area).

To determine the number of viable organisms in oral tissues, the tongue was longitudinally dissected in three equal pieces and the central portion was processed for colony forming unit (CFU) quantification at the time of sacrifice. The tissue was weighed, rinsed with phosphate buffered saline and then homogenized using a tissue homogenizer. Serial dilutions were plated onto Sabouraud dextrose agar plates containing 10 mg/ml chloramphenicol (Sigma, USA) and plates were incubated at 30°C for 48 h. Results were expressed as log CFU counts/g of tissue. One portion of the longitudinally dissected tongues was fixed with 10% buffered formalin and embedded in paraffin. Five µm thick sections were prepared and stained with hematoxylin and eosin (H&E) and Periodic Acid Schiff (PAS) stains.

### Three-dimensional model of the oral mucosa

To investigate in vitro mucosal biofilm formation, tissue damage by C. albicans and leukocyte-mediated fungal killing in an oral-like environment we used a three-dimensional model of the oral mucosa as previously described [37]. This system is composed of 3T3 fibroblasts embedded in a biomatrix of collagen type I, overlaid by a multilayer of well-differentiated oral epithelial cells (OKF6/TERT-1). C. albicans cells (1×10^6^ yeast cells) were added to the cultures apically in 100 µl of airlift medium without FBS and antibiotics. In some experiments, after 6–44 hours of co-culture mucosal tissues were formalin-fixed, embedded in paraffin and sections were stained with PAS. The extent of mucosal tissue damage was quantified at different time points by measuring extracellular leakage of LDH in the medium, using the cytotox-96 assay (Promega) as previously described [37]. Leukocyte-mediated fungal cell damage was assessed in this model as described below.

### Assessment of susceptibility to neutrophil killing

The susceptibility of the different strains to a neutrophil-like cell line (HL-60 cells) or to human freshly isolated peripheral blood neutrophils was determined by the XTT assay, as previously described [29]. Briefly, HL-60 cells were cultured in RPMI 1640 medium containing 10% fetal bovine serum and 25 mM HEPES and were induced to differentiate into neutrophil-like cells by exposure to 1.25% of dimethyl sulfoxide for 7–9 days. Neutrophils were isolated from anticoagulated blood of one healthy donor by dextran T-500 (Sigma-Aldrich, St. Louis, MO) sedimentation followed by Histopaque-1077 (Sigma-Aldrich) density gradient centrifugation. Granulocyte-erythrocyte pellet was collected, and erythrocytes were lysed by hypotonic shock. Neutrophils were washed with HBSS without Ca, Mg (Mediatech, Inc., Herndon, VA) and resuspended in RPMI1640 (Mediatech, Inc.) with 10 mM HEPES (Gibco Invitrogen, Grand Island, NY). The resulting cell preparations consisted of more than 95% of neutrophils by Wright-Giemsa stain and were more than 98% viable by trypan blue exclusion.

To perform Candida killing assays on plastic, overnight YPD broth cultures of C. albicans were resuspended in DMEM 10% fetal bovine serum and were added to 96 well plates (100 µl/well) at concentrations ranging from 10^5^ to 2×10^4^ cells/well. There was a linear relationship between viable cell number and colorimetric signal (XTT activity) in this concentration range with all strains (not shown). Immune cells were added to C. albicans at effector to target cell ratios (E∶T) ranging from 5∶1 to 1∶2. To perform Candida killing assays in an oral-like environment C. albicans cells were grown as above and added apically to the three-dimensional model of the oral mucosa (1×10^5^ yeast cells/tissue), in 50 µl of airlift medium without FBS and antibiotics. After a 4 h incubation period, differentiated HL-60 cells were added apically at an effector to target ratio of 10∶1 and incubated for 3 more hours.

After incubation of Candida with effectors at 37°C, 5% CO_2_ for 3 hours, media were aspirated and mammalian cells were lysed with sterile H_2_O. 100 µl/well of XTT solution containing Coenzyme Q0 (0.25 mg/ml XTT and 40 µg/ml coenzymeQ0) was added to each well and plates were incubated at 37°C and 5%CO_2_ for 2 hrs. Supernatants were transferred into new plates, and optical densities (OD) were measured by an Opsys Microplate Reader (Thermo Labsystems, Franklin, MA) at 450–490 nm, with a 630 nm reference filter. Antifungal activity was calculated according to the following formula: %fungal damage = (1−x/n)*100, where x is the OD450 of experimental wells (C. albicans with effectors) and n is the OD450 of control wells (C. albicans only).

### Real-time RT-PCR

To test expression of the Bcr1-regulated gene *ALS3* in an oral model of infection, we quantified expression by real-time RT-PCR after 24 h growth on the 3D model of the human oral mucosa. Briefly, media were aspirated and the collagen gel was transferred to a centrifuge tube, followed by a brief centrifugation at 4°C. Next, the transferred gel was dissolved in mammalian cell RNA extraction buffer (4 M guanidine thiocyanate, 25 mM sodium citrate, 0.5% Sarkosyl [*N*-lauroyl-sarcosine], and 0.1 M Beta-mercaptoethanol), and repeatedly passed through a 20½ gauge needle [38]. The organisms were spun in a centrifuge at 14 000×*g* at 4°C, and then snap-frozen in liquid nitrogen. Fungal RNA was isolated using the RiboPure yeast kit (Ambion, Inc.), according to the manufacturer's instructions. RNA was reverse transcribed with oligo(dT) primers using Superscript reverse transcriptase II (Invitrogen).

Primers used for measurement of transcript levels were as follows. We used the sequences described by Green et al. [39] for measuring *ALS3* RNA levels: ALS3 FOR: 5′-CCACTTCACAATCCCCATC-3′, and ALS3 REV: 5′-CAGCAGTAGTAGTAACAGTAGTAGTTTCATC-3′. We used sequences described by Blankenship et al. [40] for measuring control *TDH3* RNA levels: TDH3 FOR: 5′-AAATCGGTGGAGACAACAGC-3′, and TDH3 REV: 5′-TGCTAAAGCCGTTGGTAAGG-3′.

RT-PCR reaction conditions were as follows: 2× iQ SYBR Green Supermix (Bio-Rad), 1 µl of first-strand cDNA reaction mixture, and 0.1 µM of primers were mixed in a total volume of 50 µl per reaction. Real-time PCR was carried out in duplicate for each sample using the iCycler iQ real-time PCR detection system (Bio-Rad). The program for amplification included an initial denaturation step at 95°C for 5 min, followed by 40 cycles of 95°C for 45 s and 58°C for 30 s. Product amplification was detected using SYBR Green fluorescence during the 58°C step. The reaction specificity was monitored by melt-curve analysis. *TDH3* was used as a reference gene for normalization of gene expression, which was done using Bio-Rad iQ5 software (ΔΔ*C_T_* method).
